# Mapping Building-Based Spatiotemporal Distributions of Carbon Dioxide Emission: A Case Study in England

**DOI:** 10.3390/ijerph19105986

**Published:** 2022-05-14

**Authors:** Yue Zheng, Jinpei Ou, Guangzhao Chen, Xinxin Wu, Xiaoping Liu

**Affiliations:** 1 Guangdong Key Laboratory for Urbanization and Geo-Simulation, School of Geography and Planning, Sun Yat-sen University, Guangzhou 510275, China; zhengy235@mail2.sysu.edu.cn (Y.Z.); oujinpei3@mail.sysu.edu.cn (J.O.); wuxx33@mail2.sysu.edu.cn (X.W.); liuxp3@mail.sysu.edu.cn (X.L.); 2 Faculty of Architecture, The University of Hong Kong, Pokfulam, Hong Kong SAR, China; 3 Southern Marine Science and Engineering Guangdong Laboratory (Zhuhai), Zhuhai 519000, China

**Keywords:** CO_2_ emissions, linear regression analysis, radiance-calibrated nightlight, building-based

## Abstract

The spatiotemporal inventory of carbon dioxide (CO_2_) emissions from the building sector is significant for formulating regional and global warming mitigation policies. Previous studies have attempted to use energy consumption models associated with field investigations to estimate CO_2_ emissions from buildings at local scales, or they used spatial proxies to downscale emission sources from large geographic units to grid cells for larger scales. However, mapping the spatiotemporal distributions of CO_2_ emissions on a large scale based on buildings remains challenging. Hence, we conducted a case study in England in 2015, wherein we developed linear regression models to analyze monthly CO_2_ emissions at the building scale by integrating the Emissions Database for Global Atmospheric Research, building data, and Visible Infrared Imaging Radiometer Suite night-time lights images. The results showed that the proposed model that considered building data and night-time light imagery achieved the best fit. Fine-scale spatial heterogeneity was observed in the distributions of building-based CO_2_ emissions compared to grid-based emission maps. In addition, we observed seasonal differences in CO_2_ emissions. Specifically, buildings emitted significantly more CO_2_ in winter than in summer in England. We believe our results have great potential for use in carbon neutrality policy making and climate monitoring.

## 1. Introduction

Atmospheric carbon dioxide (CO_2_) levels are at their highest in recent history. Increasing CO_2_ emissions have exacerbated the greenhouse effect and led to global warming, which in turn has spurred a series of environmental issues, such as sea level and temperature rise, increased incidence of extreme weather, and other potential hazards to global public health [1,2,3]. According to the Fifth Assessment Report of the Intergovernmental Panel on Climate Change (IPCC), human activities have been the main drivers of global warming since the Industrial Revolution of the mid-20th century [4]. As hubs for human social and economic activities, cities are reported to emit 71–76% of the total global energy-related carbon emissions, of which the building sector occupies approximately one-third [5,6]. For example, buildings account for more than 70% of the total energy consumption and CO_2_ emissions in some major cities in the USA [7], approximately 59% of electricity consumption in the European Union [8], and over 60% of carbon emissions in Hong Kong, China [9]. With continuous advancement of urbanization and improvement of living standards, CO_2_ emissions from buildings are projected to increase, which warrants closer attention [10,11]. Therefore, emission reduction in the building sector is considered critical for effectively controlling the growth of CO_2_ emissions from rapid urbanization. For emission reduction goals, it is essential to understand and assess CO_2_ emission patterns in the building sector and investigate their spatiotemporal distributions, thus providing a foundation for the development of low-carbon cities.

For several decades, many domestic and international researchers have conducted valuable investigations on the assessment of CO_2_ emissions from buildings. One of the most representative studies is the sustainable building assessment technical system proposed by the German Sustainable Building Association, which calculated the total life cycle carbon emission inventory of a building, including the production and construction, operation, maintenance, renewal, demolition, and reuse phases. In addition to the life-cycle-based method, some research institutions have proposed generic models for estimating energy consumption in the operational phase of existing buildings, such as the DeST model established by the School of Architecture, Tsinghua University [12,13], the Quick Energy Simulation Tool (eQuest) software [14] and EnergyPlus [15] developed by the US Department of Energy, and the CitySim operated by Swiss federal Institute of Technology in Lausanne [16]. Although the above models and software can assess energy consumption and CO_2_ emissions by simulating a building’s environment and equipment systems, most of them can only be applied to a single building. Such methods not only ignore the calculation of CO_2_ emissions in buildings at the city scale and above, but they also fail to provide an in-depth spatial analysis of the urban carbon cycle and heat island effects. Gurney et al. [17] used the eQuest model to estimate the energy consumption characteristics of buildings in Indianapolis and estimated the spatial and temporal distribution of carbon emissions for each building in the city. However, the building energy simulation tool requires complex field surveys and statistics, such as meteorological data and basic parameters of thermal disturbance, as model input parameters. In assessing the distributions of CO_2_ emissions from urban buildings, such approaches, which require large-scale field investigations and statistical data, are costly and time-consuming, thereby limiting their application and efficiency in large regions.

The other widely used approach for CO_2_ assessment at the citywide and larger scales is the top-down downscaling method that distributes emission sources from a large geographic region to smaller regions. Most existing downscaling methods allocate CO_2_ based on spatial proxies such as night-time light imagery and population grid data. For example, on the basis of population data and administrative division data, Andres et al. [18] established the spatial distribution data of CO_2_ emissions, with a spatial resolution of 1°, from 1950 to 1990, and analyzed the growth characteristics of carbon dioxide emissions in different regions. Additionally, Oda and Aksyutov [19] constructed an open-source data inventory of anthropogenic CO_2_ (ODIAC) with a spatial resolution of 1 km, based on the relationship between night-time light images and the total carbon emissions of countries. Liu et al. [20] used a linear relationship to calculate global daily residential CO_2_ emissions during COVID-19 pandemic at the country scale. Zhao et al. [21] downscaled building energy consumption carbon emissions at 1 km resolution by machine learning. These spatial proxies helped rationally allocate carbon emissions from buildings. For example, the Emissions Database for Global Atmospheric Research (EDGAR), developed by the European Commission Joint Research Centre (EU-JRC) and the Netherlands Environmental Assessment Agency (PBL), is one of the most representative inventories of carbon emissions, which utilizes energy and manufacturing facilities locations, road networks, the density of human and animal population, and a number of other spatial proxies. EDGAR provides spatially gridded data of global carbon emissions from buildings and other sectors of agricultural, transport, power, residential, industrial, and manufacturing [22].

The global carbon grid includes global 0.1° × 0.1° CO_2_ emission inventories in 2019 for the residential, power, industrial, transport, shipping, and aviation sectors with a calculation framework which integrates multiple data flows [23,24,25]. However, the existing inventories of CO_2_ emissions at large scales are generally operated within a grid unit (e.g., 1 km or 0.1° resolution), which may contain hundreds or thousands of buildings. Owing to the different types of human activities occurring in buildings (including studying, working, entertainment, and catering), the spatial distributions of CO_2_ emissions from different buildings within the same grid might be significant differences. The grid-based inventories of CO_2_ emissions cannot distinguish CO_2_ emissions between different buildings, which may limit the assessment of the exact distributions of CO_2_ emissions. Therefore, it is necessary to construct building-based spatial distributions of CO_2_ emissions.

Further, the emission levels from individual buildings can vary over time due to changes in residential activities’ intensity. Specifically, the carbon emissions in a building differ significantly throughout the year as the different energy needs for heating/cooling are affected by seasonality [20]. However, most current analysis methods and datasets of CO_2_ emissions from individual buildings are based on a yearly basis or have a time lag of at least one year, making it difficult to analyze the temporal dynamics of CO_2_ emissions accurately. To address this issue, some studies have attempted to establish reliable near-real-time data on carbon emissions. For example, EDGAR provides spatial maps of global carbon emissions with yearly, monthly, and hourly data [22]. In addition, Liu et al. [26] estimated near-real-time global daily CO_2_ emissions on a 0.1° grid from sectors including power generation, industry and cement production, ground transportation, and commercial or residential construction. Unfortunately, such studies on the temporal dynamics of CO_2_ emissions have only focused on the emissions within grid units and ignored the detailed spatial characteristics of buildings. As the building-based CO_2_ emissions in large regions have rarely been analyzed and discussed, mapping building-based CO_2_ emissions with temporal dynamics quickly and accurately remains a challenge.

With the emergence of ambitious climate policies and mitigation efforts [27,28], a reliable dataset of high-resolution spatiotemporal distributions of CO_2_ emissions from buildings in large regions is needed to inform legislation. Based on these considerations, we attempted to integrate night-time imagery and building data to construct a monthly inventory of building-based CO_2_ emissions using a case study in England, United Kingdom (UK). We first collected several datasets, including building data, Visible Infrared Imaging Radiometer Suite (VIIRS) night-time light imagery, and building-sector CO_2_ emissions derived from EDGAR. We then built linear regression models for estimating the relationship between CO_2_ emissions and two factors (building volume and night-time light) at the county level in each month. We selected the model with the best performance to help construct monthly CO_2_ emission inventories for more than 11 million individual buildings in England. This study aimed to inform the development of a low-carbon city and promote sustainable development by analyzing the detailed spatiotemporal distribution of building-based CO_2_ emissions in England.

## 2. Materials and Methods

### 2.1. Study Area

**Figure 1:**
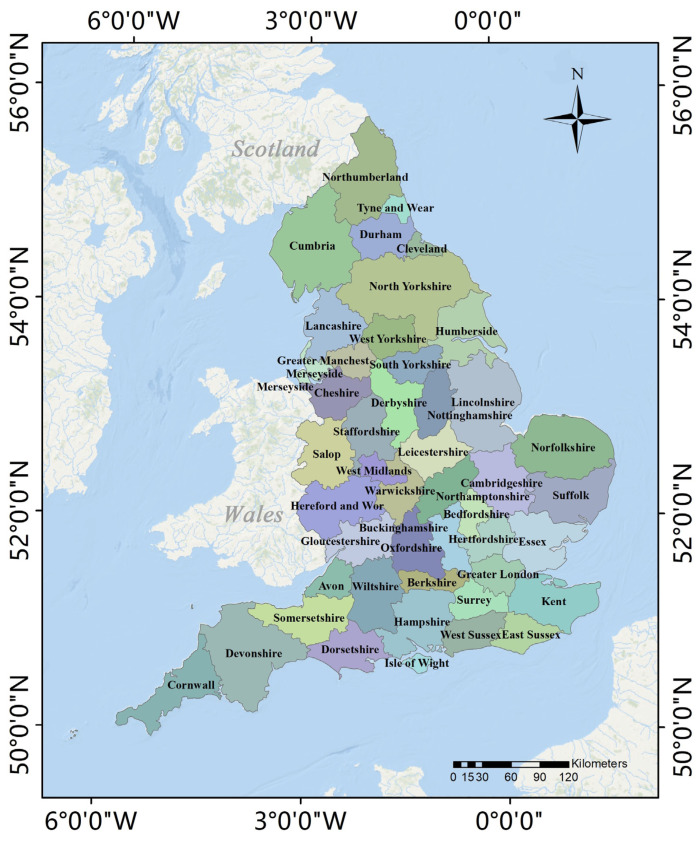
The location and spatial distributions of 46 counties in England.

England, the main body of the United Kingdom, Great Britain, and Northern Ireland, was selected as the research area for this study (Figure 1). England has a total area of 130,279 km^2^, a population of approximately 56 million, and is located in the southeast of the island of Great Britain, bordering Wales in the west and Scotland in the north. England was one of the first countries globally to promote industrialization and urbanization; therefore, it is currently viewed as a fully developed nation. Moreover, England has become a highly developed capitalist region with an urbanization rate of more than 90% [29]. England has also been a global leader in the development of low-carbon buildings possessing a sound regulatory framework and mature, low-energy technologies. At the beginning of the 21st century, the British government proposed a low-carbon economic plan and successively launched the Low Carbon Building Program and Renewable Heat Incentive [30]. According to the report of 2020 UK Greenhouse Gas Emissions, England’s total carbon emission was 320 million tons, which has since fallen to levels not seen since 1888 during the first Industrial Revolution and that are 49.7% lower than they were in 1990. To further control carbon emissions, the UK passed the Climate Change Act in 2019, in which the government committed to net-zero greenhouse gas emissions by 2050. Studying England’s approach to reducing emissions has significance for other countries and regions of the world; therefore, England is a suitable subject for studying the spatiotemporal distributions of CO_2_ emissions from buildings.

### 2.2. Overall Framework

**Figure 2:**
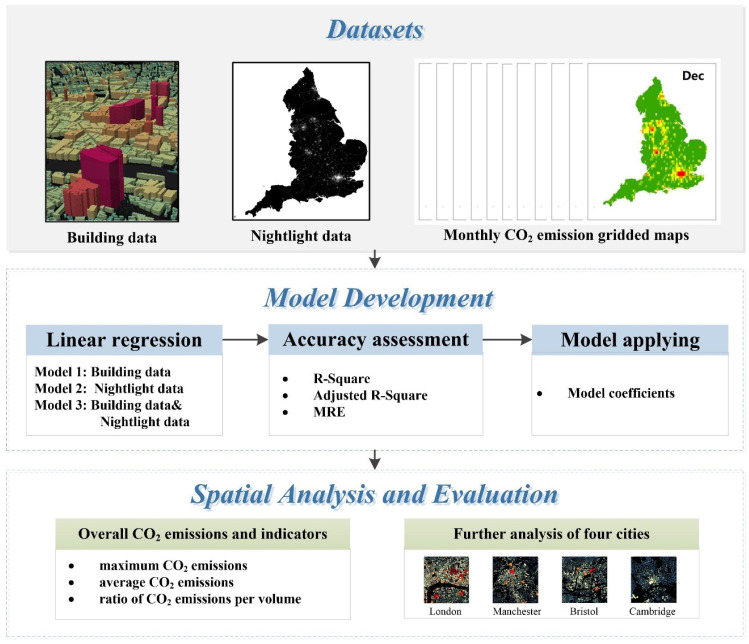
Overall flowchart for estimating building-based CO_2_ emissions.

We used linear regression models to estimate monthly building-based CO_2_ emissions in England in 2015 based on emission data from EDGAR and two factors (night-time lights and building data). The overall framework of this study is illustrated in Figure 2, which can be broken up into three parts: (1) dataset preparation, in which we collected and processed the relevant data as model variables; (2) model development, wherein we constructed three linear regression models with different factors, verified the model performances using three accuracy assessment indicators, and applied the model with the best performance to estimate monthly building-based CO_2_ emissions; (3) spatial analysis and evaluation, including analyzing the spatial distributions and seasonal characteristics of building-based CO_2_ emissions in England using multiple metrics and conducting a further analysis on four typical cities.

### 2.3. Data Preparation and Processing

#### 2.3.1. Monthly CO_2_ Emissions Data from EDGAR

**Figure 3:**
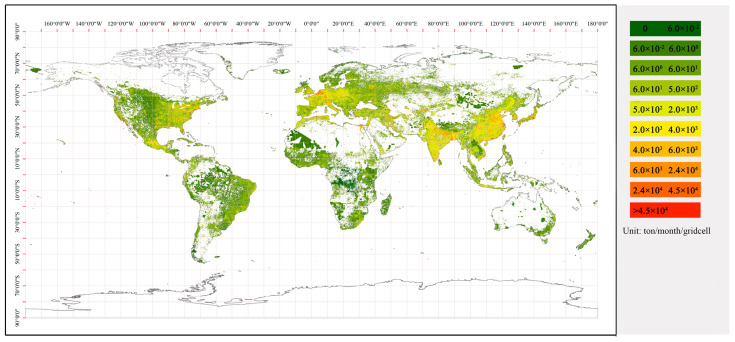
EDGAR gridded map of building-sector CO_2_ emissions in January.

The EDGAR dataset was provided by the EU-JRC and the PBL. It contains global gridded carbon emission data from various sectors at a 0.1° × 0.1° resolution, including agriculture, power, transport, residence, industry, manufacturing, and more [22,31,32,33]. The CO_2_ emissions data of EDGAR were derived from the national CO_2_ statistic reported by the Global Carbon Project and broken down to IPCC-relevant source-sector levels. EDGAR is a well-known dataset for its reliability and accuracy of data quality and thus has been widely applied in various environmental research and management [20,26]. The monthly CO_2_ emissions for 2015 in the energy sector for buildings originating from EDGAR (https://edgar.jrc.ec.europa.eu/gallery?release=v50&substance=CO2_org_short-cycle_C%20&sector=RCO, accessed on 4 May 2020)were used in this study as the ideal input data for building-based CO_2_ emission estimates (Figure 3).

The gridded maps from EDGAR of building-sector CO_2_ emissions are spatial data arranged in 0.1° × 0.1° grid cells; therefore, the emission value in each cell should be aggregated into the unit of the county in England to build regression models at county scale. To reasonably aggregate CO_2_ emissions from buildings in 46 counties, we clipped the gridded maps with the boundaries of each county, some of which were clipped into multiple sections. With regard to these incomplete grids, the CO_2_ emissions were reasonably allocated by comparing the area of each clipped part to that of the complete grid. When computing the CO_2_ emissions of a county, the total value of the emissions was equal to the emission values of the complete grids and the emission values of these parts within the county. The calculation formula is shown in Equation (1):

(1) $$E_{k}=\sum_{a=1}^{m}F_{a}+\sum_{b=1}^{n}(F_{b}\times \frac{S_{kb}}{S_{b}})$$

where $E_{k}$ is the total value of CO_2_ emissions in county *k*; *F_a_* is the value of CO_2_ emissions in grid *a*; *m* and *n* represent the number of complete and incomplete grids in this county, respectively; $S_{b}$ is the area of grid *b*; and $S_{kb}$ is the area of grid *b* occupied by county *k*.

#### 2.3.2. Building Data

**Figure 4:**
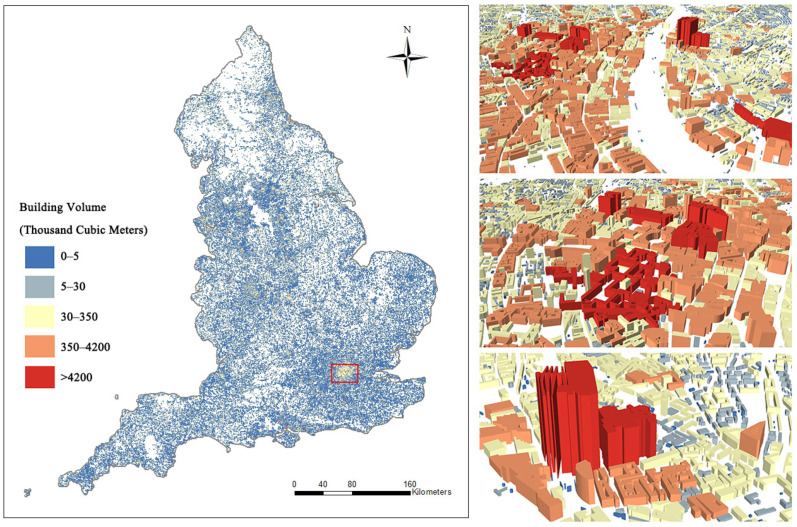
The spatial distribution of building volumes in England.

Buildings are an important unit of urban three-dimensional structures that significantly influence land use type, development of infrastructure, efficient use of natural resources, and CO_2_ emissions [34,35,36]. The building volume, which refers to the volume of a building in space, is considered an important driving force accelerating urban carbon emissions [37]; thus, building volume is considered an important indicator of urban energy efficiency and CO_2_ emission levels. To calculate the volume of each building in England, we collected building data on the building footprint and height data provided by Emu Analytics (http://buildingheights.emu-analytics.net, accessed on 4 May 2020). The building footprint data were obtained from the Ordinance Survey Open Map (https://www.ordnancesurvey.co.uk/business-government/tools-support/open-map-local-support, accessed on 4 May 2020), which includes nearly 12 million publicly available building footprints in England, while the building height was calculated from 1 m resolution light detection and ranging (LiDAR) images. The calculations for building height are shown in Figure 4.

#### 2.3.3. NPP-VIIRS Night-time Lights Imagery

Night-light images, which capture the near-infrared electromagnetic wave signals emitted from a given surface, can provide a unified, continuous, and timely measurement of human activities. In recent years, night-time light data, mainly derived from the Defense Meteorological Satellite Program’s Operational Linescan System (DMSP-OLS) and the Visible Infrared Imaging Radiometer Suite on the Suomi National Polar-orbiting Partnership satellite (NPP-VIIRS), have been widely used to assess city scope, urbanization processes, population distributions, socioeconomic dynamics, and CO_2_ emission distributions [38,39,40,41,42]. Compared with DMSP-OLS, NPP-VIIRS have higher spatial resolution (500 m), no light saturation, and higher light capture sensitivity, especially in the acquisition of low-intensity light emitted by human activities and the detection of electric lighting on the Earth’s surface [43]. Currently, NPP-VIIRS have proven to be more appropriate for estimating the spatiotemporal distributions of carbon emissions [44]. Therefore, in this study, the monthly NPP-VIIRS are used to estimate CO_2_ emissions from buildings. It is important to note that the NPP-VIIRS night-time light data used in this study are cloud-free observation monthly images, provided by the National Oceanic and Atmospheric Administration (NOAA), that have already eliminated stray light pollution and background noise.

**Figure 5:**
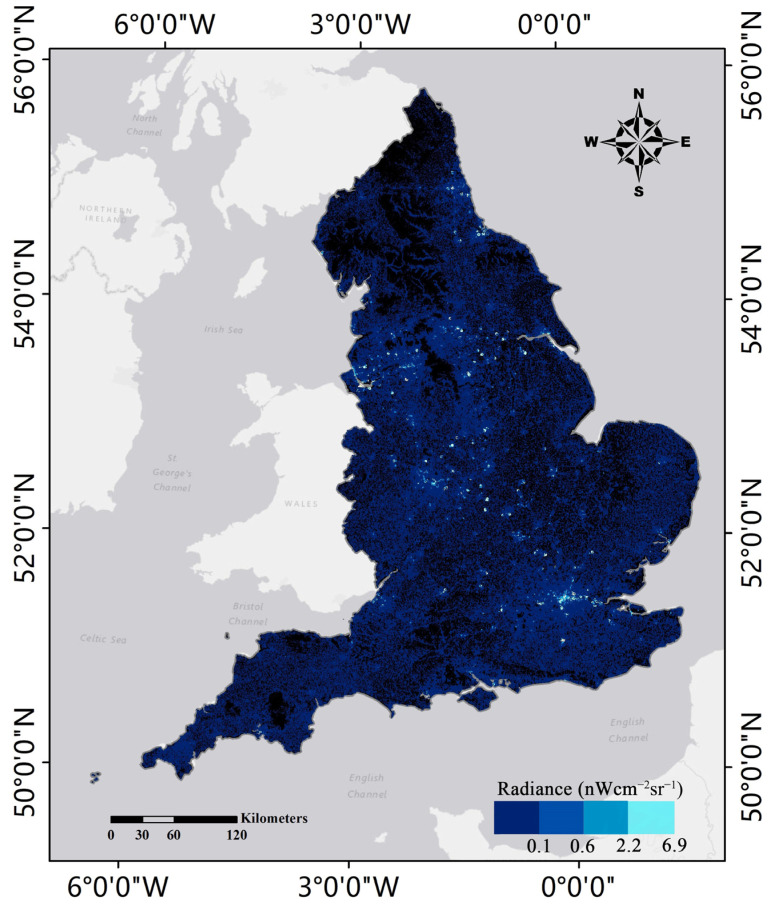
The distribution of single building’s nightlight value in England in January.

To obtain the total night-time light generated only by the buildings, the nightlight value of each building was calculated by the proportion of the building’s footprint area to the area of a night-time light grid. The specific formula for calculating the night-time light value of a building is shown in Equation (2):

(2) $$L_{kj}=L_{t}\times \frac{S_{kj}}{S_{t}}$$

where $L_{kj}$ is the night-time light value of building *j* in county *k*, $L_{t}$ is the total night-time light value in grid *t*, where the building is located, $S_{kj}$ is the footprint area of building *j* in county *k,* and $S_{t}$ is the area of grid *t*. According to Equation (2), the night-time light value of a single building in each month was calculated and collected. Figure 5 shows the distributions of night-time light values for each building in England in January.

### 2.4. Linear Regression Analysis

As a widely used statistical analysis method for modeling the quantitative relationship between two or more variables, linear regression has proven to be reliable to assess and predict CO_2_ emissions [45,46,47]. For example, Seyed [48] attempted to project Iran’s CO_2_ emissions through 2030 by developing a multiple linear regression model. Similarly, Samuel and Phebe [49] used a linear regression approach to examine the relationships between CO_2_ emissions, energy use, GDP, and population in Ghana from 1971 to 2013. In this study, we used linear regression to analyze the relationship between CO_2_ emissions, night-time light imagery, and building data. We elected to build different linear regression models for each month due to seasonal variation in CO_2_ emissions.

To investigate the significance of different factors to building CO_2_ emissions, we also constructed three linear regression models to analyze the relationship between building data, building night-time light data, and CO_2_ emissions from buildings on the county scale. The total building volumes and night-time light values for each county can be obtained by adding up the individual building volumes and individual night-time light values for all buildings within the county. Model 1 was based on the correlations between the total volume of all buildings and CO_2_ emissions from buildings in 46 counties. Model 2 was based on the correlations between the total VIIRS night-time light values and CO_2_ emissions from all buildings. In Model 3, both night-time lights and building volume were used as explanatory variables. The equations for the three Models (3)–(5) are presented as follows:

(3) $$\mathrm{Model}\;1:f_{ki}=a_{i}\times \sum V_{kj}$$

(4) $$\mathrm{Model}\;2:f_{ki}=b_{i}\times \sum L_{kji}$$

(5) $$\mathrm{Model}\;3:f_{ki}=a_{i}\times \sum V_{kj}+b_{i}\times \sum L_{kji}$$

where $f_{ki}$ is the total CO_2_ emissions from buildings in county *k* in month *i*; $a_{i}$ and $b_{i}$ are the model coefficients in month *i*; $\sum V_{kj}$ is the sum of the volume of all buildings in county *k*; and $\sum L_{kji}$ is the total night-time light values of all buildings in county *k* of month *i*.

### 2.5. Evaluation Analysis

Referring to previous studies, we introduced goodness-of-fit (*R*^2^), adjusted *R*^2^, mean relative error (MRE), and root-mean-square error (RMSE) of linear regression to evaluate the performance and accuracy of each month’s model in emission estimation. The *R*^2^ values ranged from 0 to 1. We also applied the MRE and RMSE to evaluate the agreement between the estimated emission and the true emissions. The formulas (Equations (6)–(9)) for our statistical analyses are as follows:

(6) $$R^{2}=1-\frac{\sum {\left(y_{i}-{\hat{y}}_{i}\right)}^{2}}{\sum {\left(y_{i}-\bar{y}\right)}^{2}}$$

(7) $$\mathrm{Adjusted}\;R^{2}=1-\frac{\left(1-R^{2})(1-n\right)}{\left(n-k-1\right)}$$

(8) $$\mathrm{MRE}\;=\frac{1}{n}\sum \left|\frac{y_{i}-{\hat{y}}_{i}}{y_{i}}\right|\times 100\%$$

(9) $$\mathrm{RMSE}\;=\sqrt{\frac{1}{n}\sum_{i=1}^{n}{\left(y_{i}-{\hat{y}}_{i}\right)}^{2}}$$

where *n* is the sample size; $y_{i}$ is the actual statistical data, ${\hat{y}}_{i}$ is the estimated emission value; $\bar{y}$ is the mean of actual CO_2_ emissions; and *k* is the number of independent variables.

### 2.6. Multicollinearity Diagnosis

We conducted a multicollinearity test to identify any multicollinearity among the different variables. Multicollinearity refers to the fact that linear regression models are distorted because of the highly correlated relationships between explanatory variables. The variance inflation factor (VIF) is a widely used metric to measure the degree of multicollinearity of explanatory variables in multiple linear regression models [50]. VIF was calculated using Equation (10):

(10) $$\mathrm{VIF}=\frac{1}{1-R_{i}^{2}}$$

where *R_i_* is the negative correlation coefficient of independent variable *X_i_* for the regression analysis of the remaining independent variables.

### 2.7. Lorenz Curve and GINI Coefficient

Inspired by the income Lorenz curve, the Lorenz curve has been introduced to the field of energy consumption by some scholars, and it proved to be useful to evaluate the inequality of building energy [51]. The Gini coefficient, which is closely related to the Lorenz curve, is a commonly used indicator to measure the income gap of residents in a country or region [52]. Some researchers also use an environmental Gini coefficient to access the degree of allocation equality for various environmental issues, such as carbon emissions [53]. In order to evaluate the allocation results of CO_2_ emissions from buildings in England, we substitute the allocated emissions for income, and the number of buildings for the population. The environmental Gini coefficient can be calculated as follows:

(11) $$S_{B}=\sum_{i=1}^{n}\frac{\left(x_{i}-x_{i}-1)(y_{i}+y_{i}-1\right)}{2}$$

(12) $$\mathrm{Gini}=\frac{S_{A}}{S_{A}+S_{B}}=\frac{S_{A}}{0.5}=2S_{A}=1-2S_{B}$$

where *S_A_* is the area between the equality line and the Lorenz curve, and *S_B_* is the area under the Lorenz curve. *x_i_* is the cumulative share of numbers of building up to county *i*, and *y_i_* represents the cumulative share of building CO_2_ emissions quotas up to county *i*.

## 3. Results and Discussion

### 3.1. Comparison of Model Performance

The statistical performance indicators for the three models are listed in Table 1. For Model 1, the average values of *R*^2^ and adjusted *R*^2^ in each month were 0.862 and 0.859, respectively, while the MRE was approximately 34% and RMSE ranged from 24,000 to 90,000 tons. For Model 2, the average value of *R*^2^ in each month was 0.858, and the average value of the adjusted *R*^2^ was 0.855, while the MRE ranged from 46.62% to 50.34%. For Model 3, the values of *R*^2^ and adjusted *R*^2^ for each month were above 0.89, and *R*^2^ reached the highest value of 0.911 in July, while the MRE for each month was below 26%. The RMSE of the model was the lowest of the three models, ranging from 20,000 to 75,000 tons. Evidently, Model 3 performed best among the three models, which indicates that both building volume and night-time lights are significant factors in estimating the CO_2_ emissions from buildings, and considering both factors can greatly improve model performance.

**Figure 6:**
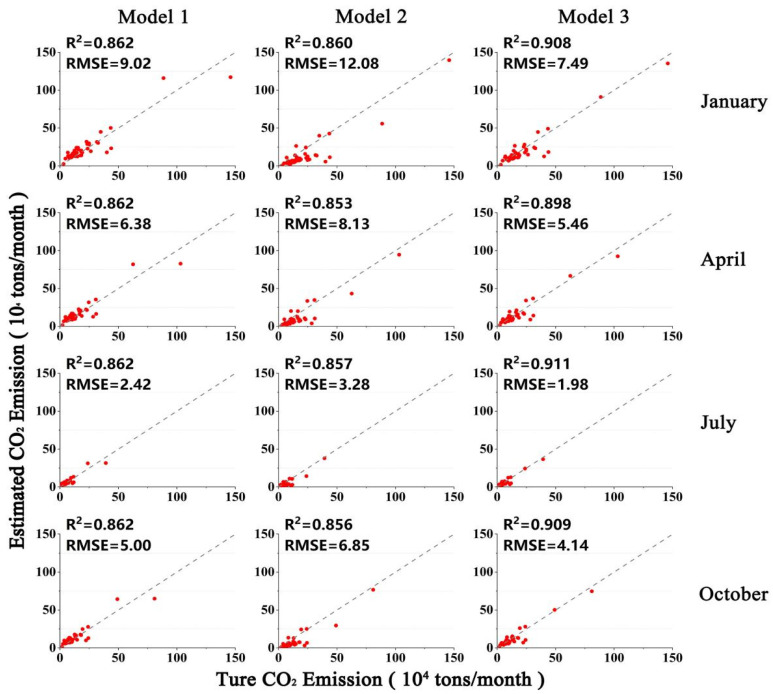
Scatter-plot of true and estimated CO_2_ emissions from buildings at county scale for three models. Each point in a scatterplot represents a county, and there are 46 points in each scatterplot.

To verify the effectiveness of the three models in estimating CO_2_ emissions from buildings, we constructed scatterplots of county-scale true and estimated CO_2_ emissions for regression Models 1–3, as shown in Figure 6. Each model type was tested in January, April, July and October. The horizontal axis represents the actual CO_2_ emissions from buildings in each county counted from the EDGAR grid map, and the vertical axis represents the CO_2_ emissions from buildings estimated by the three models. For Model 1, the scatter points are more evenly distributed on both sides of the trend line, though there was considerable under- or over-estimation of CO_2_ emissions in a few counties. For Model 2, most of the scatter points are below the one-to-one diagonal, and the RMSE of Model 2 was also the largest among the three models, suggesting that CO_2_ emissions were severely underestimated. In Model 3, the points are adjacent to the one-to-one diagonal, which is the best fit of the three models. The *R*^2^ and RMSE values further verify that the simulated values in Model 3 were closest to the predicted values. In July, the *R*^2^ of Model 3 was enhanced from 0.862 to 0.911 when compared to the Model 1, and from 0.857 to 0.911 when compared to the Model 2, indicating that this model has the best fitting accuracy and highest feasibility. Moreover, the results also indicate that Model 3 maintains a degree of stability in estimating results in different months, with the value of *R*^2^ varying only between 0.89 and 0.91.

Compared with the results from Model 1, which was constructed based only on building volume data, or Model 2, which was based only on night-time light as independent variables, regression models (Model 3) taking both explanatory variables into consideration together exhibit better performance and more stability, implying that both building volume and night-time lights are important factors for estimating CO_2_ emissions from buildings, and incorporating both variables can significantly improve the explanatory capacity of the model. Therefore, Model 3 is the most reliable and reasonable model for mapping the spatial distributions of building-based CO_2_ emissions. Subsequently, according to the regression model coefficients, Model 3 was selected for the estimation of the monthly CO_2_ emissions of each building in England.

To further evaluate the results, a multicollinearity diagnosis for Model 3 was necessary to verify the multicollinearity problem at the county scale. Table 2 presents the multicollinearity test results for each variable. The VIF for two variables in each month is smaller than 10, demonstrating that weak correlations exist among these variables, and it is feasible to establish regression Model 3 with building data and nightlight data.

The monthly coefficients of Model 3 and the evaluation results are presented in Table 2. For the model coefficients of each month, both $a_{i}$ and $b_{i}$ show significant seasonal variation, suggesting that the changes in night-time lights and building data in winter have a greater impact on CO_2_ emissions than those in summer. The f-test significance results for the models in each month were 0, indicating that all models have a statistically significant predictive capability for CO_2_ emissions from buildings. For the *t*-test statistics, the significance values for night-time light data and building data in each month were less than 0.01, meaning that both variables can significantly affect the estimation of CO_2_ emissions.

Thereafter, based on the coefficients obtained from Model 3, we can disaggregate the CO_2_ emission data from the EDGAR grid map for more than 11.86 million buildings in England from January to December by using Formula (13):

(13) $$f_{ij}=a_{i}\times V_{j}+b_{i}\times L_{ij}$$

where $f_{ij}$ is the CO_2_ emissions from building *j* in month *i*; $a_{i}$ and are the model coefficients in month *i*; $V_{j}$ is the volume of building *j*; and $L_{ij}$ is the night-time light values of building *j* in month *i*.

### 3.2. National-Scale CO_2_ Emissions Analysis

**Figure 7:**
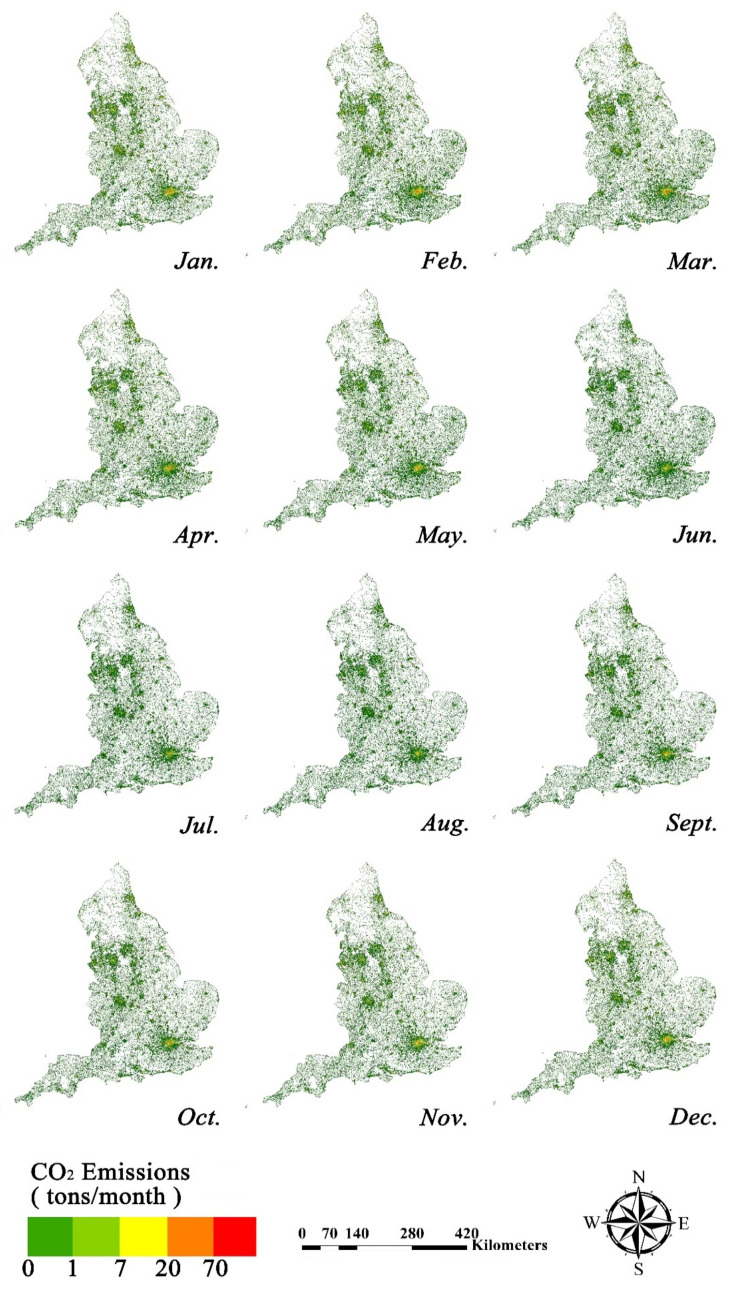
England monthly CO_2_ emissions map.

Based on the models and coefficients in Table 2, we successfully downscaled the CO_2_ emissions from the EDGAR grids to individual buildings by coupling the building data and night-time light data. The spatiotemporal variations in CO_2_ emission data from buildings at the national scale are presented in Figure 7. CO_2_ emissions from buildings in England showed obvious seasonality, being significantly higher in winter than in summer. EDGAR grid maps showed that, in winter, there were many areas scattered with red buildings, where the CO_2_ emissions from a single building exceeded 70 tons a month, whereas in summer, most urban centers are only scattered with orange buildings, where the CO_2_ emissions were less than 70 tons a month. According to one report [54], the average temperature in England in January is 4–7 °C, and the average temperature in July is 13–17 °C. The fact that CO_2_ emissions from buildings are significantly higher in winter than in summer may be due to winter heating, which causes more energy consumption.

From the perspective of spatial distribution, there is obvious spatial heterogeneity in CO_2_ emissions from buildings. As seen in Figure 7, most buildings are rendered green, which indicates that almost all the buildings emit less than 1 ton of CO_2_ per month throughout the year. Only some counties, such as Greater London, Greater Manchester, and the West Midlands, contain red buildings whose monthly CO_2_ emissions are much higher. Especially in the CBD areas, owing to frequent socio-economic activities, higher populations, higher density of buildings, larger building volumes, and other reasons, CO_2_ emissions from buildings are significantly higher than those from other areas. Therefore, the distribution of CO_2_ emissions from buildings are seemingly affected by the distribution of large cities. Furthermore, the spatial distribution of CO_2_ emissions from buildings has a certain continuity, as buildings with high CO_2_ emissions are always clustered and the CO_2_ emissions of adjacent buildings have comparatively small differences. Overall, CO_2_ emissions showed a decreasing trend from city centers to the surrounding areas.

We further adopted several statistical metrics, including the monthly maximum CO_2_ emissions (Max CO_2_), the monthly average CO_2_ emissions (Ave CO_2_), and the ratio of CO_2_ emissions per volume (CO_2_/Vol), to quantitatively evaluate the overall CO_2_ emissions from buildings at national scale (Table 3). For the specific implication of each metric, max CO_2_ refers to the maximum CO_2_ emissions from individual buildings during a month. Ave CO_2_ is the average CO_2_ emission from individual buildings during a month, which can reflect the overall situation every month. Another crucial measurement, CO_2_/Vol, refers to the monthly CO_2_ emissions produced per unit volume from buildings, which also indicates the CO_2_ emission efficiency of buildings in different regions during different months.

The results show that CO_2_ emissions from buildings vary significantly between different buildings and by month. A single building can emit up to 3682 tons of CO_2_ in January and 894 tons in August (more than four times the difference), which exceeds the average CO_2_ emissions by nearly 4000 times. For CO_2_/Vol, the ratio of CO_2_ emissions per volume is 0.152 kg/m^3^ in January, which is 3.7 times higher than the ratios in July and August.

### 3.3. County-Scale CO_2_ Emissions Analysis

**Figure 8:**
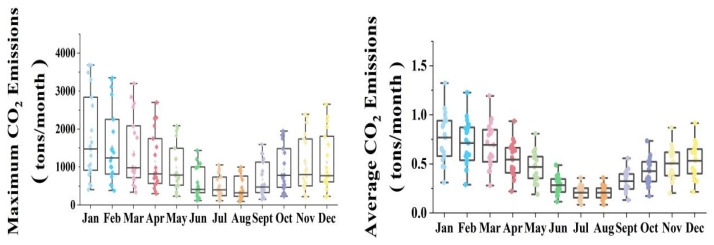
Boxplot of maximum and average CO_2_ emissions. Each point represents the average or maximum CO_2_ emissions of a county. The five horizontal lines from top to bottom represent the largest observed value, the 75% quantile, the median, the 25% quantile, and the smallest observed value, respectively. Points outside the horizontal lines at either end are outlier points.

We obtained the maximum and average CO_2_ emissions from buildings for each county in England (Figure 8). We found that both maximum and average CO_2_ emissions from buildings varied significantly in different counties and months. Clear “V” shapes in the boxplot indicate that the CO_2_ emissions in nearly all counties decreased from January to July and increased from August to December. In particular, the discrepancy in CO_2_ emissions between different counties was much larger in winter than in summer. It should be noted that the discrepancy in the maximum CO_2_ emissions in all months was larger than the discrepancy in the average CO_2_ emissions, which indicates that some buildings have extremely high CO_2_ emissions.

**Figure 9:**
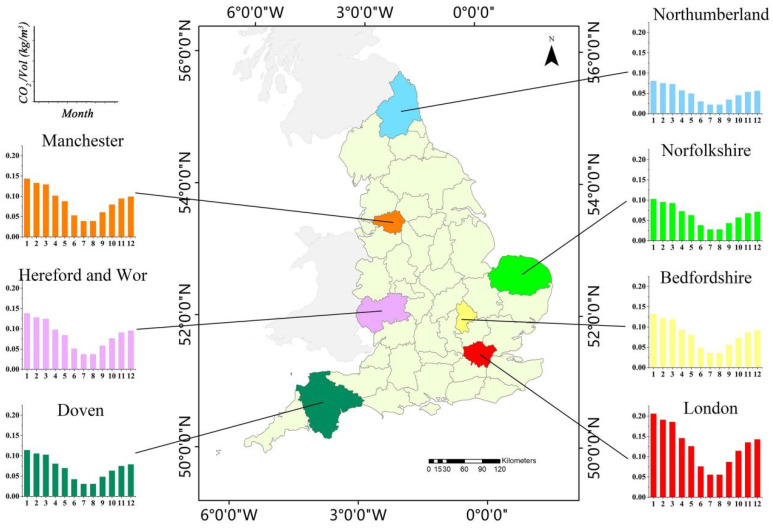
Ratio of CO_2_ emissions per volume of seven counties.

We selected seven counties, and the corresponding statistical bar graphs were drawn from the ratio of CO_2_ emissions per volume (CO_2_/Vol) for each selected county, as shown in Figure 9. The monthly changes in the CO_2_/Vol of buildings in each selected county have the same “V-shaped” trend, representing slight declines from January to July, reaching the lowest value in July and August, and subsequently increasing from September to December. In addition, the absolute ratio of CO_2_/Vol varied greatly among the different regions. For instance, as a typical metropolis, the CO_2_/Vol of Greater London County was relatively high, at 0.206 kg/m^3^ in January and 0.055 kg/m^3^ in July. In Northumberland, in northern England, the CO_2_/Vol was 0.081 kg/m^3^ in January and 0.021 kg/m^3^ in July. The CO_2_/Vol of Greater London was 2.5 times higher than that in Northumberland, showing obvious regional differences.

**Figure 10:**
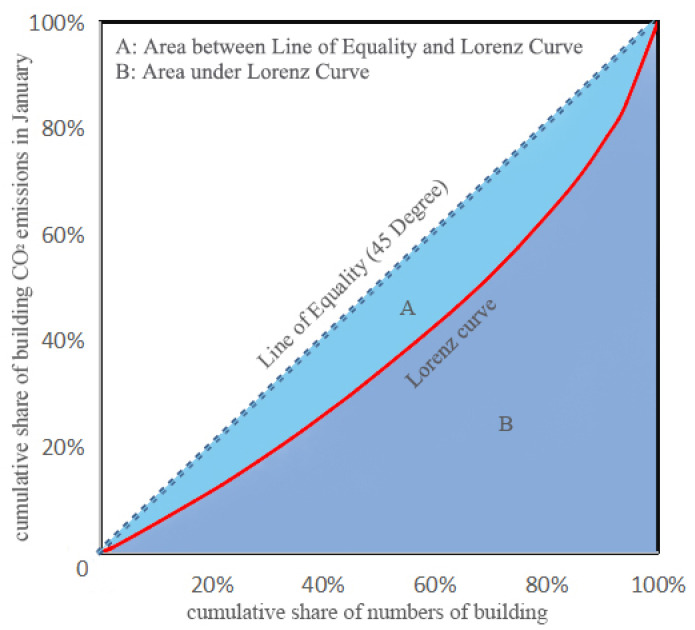
Lorenz curve for CO_2_ emission quota allocation in January.

Based on the CO_2_ emissions from each county, we plotted a Lorenz curve between CO_2_ emissions from buildings and the number of buildings in the whole England in January, as shown in Figure 10. The horizontal axis represents the cumulative share of numbers of buildings, and the vertical axis represents the cumulative share of CO_2_ emissions from buildings. The blue dotted line is the line of equality, which means the CO_2_ emissions distribution is perfectly equal. The red line is the Lorenz curve; the greater the curvature, the more unequal the distribution of building CO_2_ emissions. As the Lorenz curve shows, approximately 70% of the buildings account for 50% of the total CO_2_ emissions. The area with the highest CO_2_ emissions is Greater London, where the last 5% of buildings account for 15% of the country’s CO_2_ emissions. Based on the Formulas (11) and (12), the environmental Gini coefficient can also be calculated. The range of the Gini coefficient is from 0 to 1, and a larger Gini coefficient means a higher degree of inequality. As a watershed, 0.4 is usually considered for whether the inequality level is too high [55]. The Gini coefficient in England in January is 0.3479, which is less than 0.4, indicating that the disparity of CO_2_ emissions allocation is reasonable.

### 3.4. Building-Scale CO_2_ Emissions Analysis of Typical City

**Figure 11:**
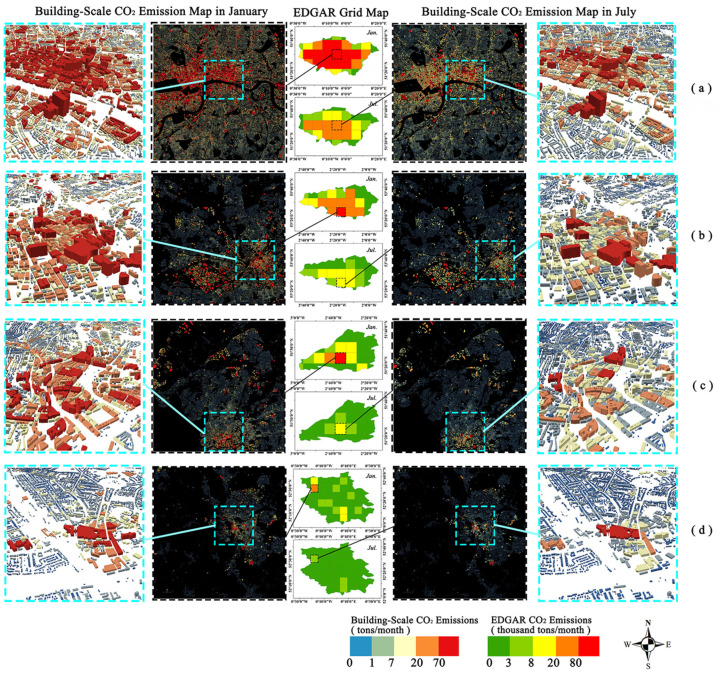
Comparison of the spatial patterns of January and July CO_2_ emissions in four regions: (**a**) Greater London, (**b**) Greater Manchester, (**c**) Bristol, and (**d**) Cambridge.

To gain a better understanding and explore the CO_2_ emission level of buildings in England, we selected urban centers of four counties located in different parts of England in January and July for further analysis. Figure 11 shows CO_2_ emission distribution maps at three different scales, including the EDGAR grid maps of four selected counties, building-based CO_2_ emissions maps within a grid cell, and detailed CO_2_ emission maps in three dimensions.

(1) London: Located in the southeast of England, London is the capital of the UK, as well as the political, cultural, and financial center [29]. London has one of the most developed city economies, most prosperous businesses, and highest living standard in the world. Figure 11a shows that the CO_2_ emissions from buildings in almost all of the Greater London area were very high in January. The detailed map shows the CO_2_ emissions from buildings in the commercial center near the Thames River, which has a large number of buildings and high building density. A large number of building CO_2_ emissions decreased significantly in July, with most buildings falling from medium or high to relatively low levels. However, many large buildings still had very high emission levels in some areas of the north shore in July, reaching more than 250 tons of CO_2_ per month. These high-carbon-emitting buildings include business centers, museums, and hotels that employ more electrical equipment, such as lifts and lights, leading to higher energy consumption.

(2) Manchester: Located in northwest England, Manchester is one of the largest metropolitan areas and one of the most important industrial centers in the UK. As shown in Figure 11b, several buildings in the central urban area had extremely high CO_2_ emissions in January, whereas most buildings in the city center emit less than 250 tons of CO_2_ per month. In July, CO_2_ emissions fell to moderate or low levels in almost all buildings, and the number of buildings with very high carbon emissions decreased. Compared with London, Manchester had significantly lower CO_2_ emissions in both January and July.

(3) Bristol: Bristol is located in Avon County, a coastal area in southwestern England. Bristol is the largest city in southwest England and houses an important commercial port and space center. As shown in Figure 11c, few buildings in the central city had extremely high CO_2_ emissions in January, with the rest of the buildings in the central city having medium or low levels, showing an obvious decreasing trend from the central city to the surrounding suburbs. In July, CO_2_ emissions fell to moderate or low levels in almost all buildings, except for a few that maintained high levels. Overall, CO_2_ emissions from buildings in Bristol were slightly lower than those in Greater London.

(4) Cambridge: Cambridge is in eastern England. Compared to other cities, Cambridge is a nonmetropolitan county with lower socioeconomic activity. In the EDGAR gridded map, depicted in Figure 11d, Cambridge had a low CO_2_ footprint across the region in January and July. As shown in the detailed view, the downtown area of Cambridge had a lower number and density of buildings and a smaller base area for each building. However, although CO_2_ emissions from buildings at Cambridge are generally low, there are still a few buildings that emit more than 250 tons of CO_2_ in January and July.

A comparative analysis of these four cities shows that there is a significant seasonal variation in CO_2_ emissions from buildings, with almost every building emitting significantly more CO_2_ in winter than in summer. Additionally, from the perspective of spatial distribution, CO_2_ emissions from buildings have an overall trend of decay from city centers to suburban areas, which means that buildings in city centers tend to have higher CO_2_ emissions. Meanwhile, there is also spatial heterogeneity as indicated by the CO_2_ emissions of some adjacent buildings, which may vary by up to a thousand times owing to large differences in building spatial structures. In addition, a comparison of the four counties in Figure 11 shows that the number of buildings in different grids varies widely, suggesting that CO_2_ emissions of the grid map are strongly related to the number of buildings in the grid and that the grid-based CO_2_ inventories are not sufficient to show the detailed spatial–temporal distributions of CO_2_ emissions for all buildings as grids with low CO_2_ emissions possibly contain a few high CO_2_-emitting buildings.

### 3.5. Policy Implications for CO_2_ Reduction

Reducing CO_2_ emissions from buildings with the requirement of green and low-carbon transformation of the economy and society is of great significance. The compilation of a CO_2_ inventory from the building sector is required to understand emission situations, establish emission baselines, verify emission trajectories, and develop efficient, feasible and viable mitigation options. This study developed linear regression models to calculate monthly building-based CO_2_ emissions. Unlike previous life-cycle-based methods or other approaches that require time-consuming field investigations and/or are based on a set of complex parameters, the mathematical method promoted in this study has been proven to be an effective way to quickly obtain building-based CO_2_ emissions over a large region by integrating only three accessible datasets, that is, gridded CO_2_ emissions from buildings, building volume data, and nightlight imagery. Therefore, this method can easily spread its use and may act as a highly efficient way to estimate the spatiotemporal distributions of building-based CO_2_ emissions when governments create low-carbon cities and sustainable development policies. More importantly, the spatial distributions of building-based CO_2_ emissions can aid in accurately identifying buildings with high CO_2_ emissions, which can offer useful support when prioritizing the priority decision of carbon reduction at a specific location.

However, the heating or cooling demands of residential and commercial buildings due to weather changes are the main reason for the large seasonal variation in CO_2_ emissions in the building sector. According to a report by the Met Office, the average temperature in the UK ranges from 4 to 7 °C in January and 13 to 17 °C in July. The rapid rise in CO_2_ emissions from buildings in England in winter is caused by extensive space heating, while a cooler climate with less cooling in summer results in lower CO_2_ emissions. Some studies have recognized that improving heating and cooling systems is an effective way to reduce CO_2_ emissions, including improving energy efficiency, increasing electrification, and creating cleaner electric grids [56]. Therefore, the fast and time-efficient CO_2_ emission estimating method with higher temporal resolution proposed in this paper can serve as a monitoring tool to provide references for policymakers in formulating and revising “decarbonization” strategy policies promptly.

### 3.6. Limitations

Further investigations are necessary to address the limitations of the present study. First, a thorough field survey is needed to obtain the actual CO_2_ emissions for buildings over a place, so as to provide true measured data to assess the accuracy of our estimated CO_2_ emissions from buildings. Second, we only considered two building factors: building volume and night-time light. Other factors, such as population distributions and energy consumption demands, might have significant impacts on CO_2_ emissions and should thus be considered. Third, fine-scale CO_2_ emission data from different types of buildings (e.g., building function, design, orientation) can provide valuable references for decision makers to develop different carbon reduction policies. However, due to the lack of a breakdown of buildings, the present study failed to consider the differences in building types when modeling building-based CO_2_ emissions in the studied region, which may introduce uncertainties into our results. Therefore, future researchers should consider the types of building when modeling CO_2_ emissions from buildings in order to identify super emitter categories.

It should be noted that the monthly CO_2_ emissions data used in our study is the EDGAR v5.0 dataset published in November 2019, which provides monthly CO_2_ emissions data from 1975 to 2015. It was the latest version available from the EU-JRC when we did the experiment. Recently, the latest version of EDGAR v6.0 was published, which provides CO_2_ emissions data for building sector from 1970 to 2018. Collecting building data and night-time light data for 2018 and using our proposed methodology to calculate monthly CO_2_ emission levels for individual buildings in 2018 and other years would be an easy and predictable work in the future.

## 4. Conclusions

In this study, we proposed a downscale methodology to quickly obtain monthly building-based CO_2_ emissions by coupling EDGAR global CO_2_ emission gridded maps, building data, and VIIRS night-time light imagery. In addition, we successfully mapped the monthly spatial distribution of CO_2_ emissions from more than 11 million buildings in England in 2015. Subsequent verification proved that our models performed well, with the results of both *R*^2^ and adjusted *R*^2^ consistently exceeding 0.89 and an MRE below 26% every month. Moreover, we conducted a detailed analysis of the spatiotemporal distribution of CO_2_ emissions from buildings in England at national, county and building scale. The analysis showed that CO_2_ emissions from buildings are spatially heterogeneous and the emissions of adjacent buildings can vary by thousands of times. In addition, there are also significant seasonal differences; the average CO_2_ emission per volume in January is 3.7 times higher than that in July.

In summary, our proposed method fills the gaps in obtaining building-based spatiotemporal distributions of CO_2_ emission in large regions. We emphasize that fine-scale CO_2_ emission data from buildings are expected to serve as an important input for various environmental and social applications, such as sustainable development and decision making. Hence, the low-cost, accurate, and reliable approach proposed in this paper for acquiring building-based CO_2_ emission data in large regions shows great potential for energy or climate impact modeling.

## Figures and Tables

**Table 1:** Comparison of emission results from Models 1–3 in different months.

Month	Model 1				Model 2				Model 3			
	*R* ^2^	Adjusted *R*^2^	MRE(%)	RMSE (10^4^ tons)	*R* ^2^	Adjusted *R*^2^	MRE(%)	RMSE (10^4^ tons)	*R* ^2^	Adjusted *R*^2^	MRE(%)	RMSE (10^4^ tons)
January	0.862	0.859	34.02	9.02	0.860	0.856	48.67	12.08	0.908	0.904	24.86	7.49
February	0.862	0.859	34.02	8.36	0.854	0.851	48.86	11.05	0.906	0.901	25.38	6.93
March	0.862	0.859	34.04	8.13	0.858	0.855	46.80	10.45	0.902	0.897	24.70	6.89
April	0.862	0.859	34.04	6.38	0.853	0.849	47.00	8.13	0.898	0.893	25.58	5.46
May	0.862	0.859	34.06	5.51	0.858	0.855	46.62	7.16	0.904	0.900	24.85	4.62
June	0.862	0.859	34.01	3.32	0.873	0.870	49.64	4.03	0.897	0.892	25.34	2.93
July	0.862	0.859	34.03	2.42	0.857	0.853	49.03	3.28	0.911	0.906	24.38	1.98
August	0.862	0.859	34.03	2.42	0.859	0.856	49.83	3.20	0.903	0.898	25.55	2.06
September	0.862	0.859	34.05	3.80	0.871	0.874	49.16	4.90	0.910	0.905	24.54	3.20
October	0.862	0.859	34.01	5.00	0.856	0.852	50.34	6.85	0.909	0.904	24.09	4.14
November	0.862	0.859	34.00	5.91	0.855	0.852	50.21	7.98	0.905	0.901	24.99	4.96
December	0.862	0.859	34.02	6.24	0.844	0.841	48.40	8.15	0.892	0.886	26.02	5.48

**Table 2:** Coefficients of models in different months.

Month	Building Data			Nightlight Data			F	Sig.
	*a_i_*	Sig.	VIF	*b_i_*	Sig.	VIF		
January	88.19	0.000	5.059	51.05	0.000	5.059	202.961	0.000
February	83.99	0.000	5.003	46.37	0.000	5.003	197.720	0.000
March	79.79	0.000	5.674	44.12	0.000	5.674	188.289	0.000
April	64.44	0.000	5.784	32.86	0.001	5.784	180.626	0.000
May	54.02	0.000	5.388	29.78	0.000	5.388	193.823	0.000
June	28.62	0.000	7.912	18.83	0.001	7.912	178.664	0.000
July	24.10	0.000	4.722	14.72	0.000	4.722	208.689	0.000
August	23.64	0.000	5.606	14.03	0.000	5.606	190.948	0.000
September	33.92	0.000	5.772	23.01	0.000	5.772	206.266	0.000
October	49.90	0.000	4.832	28.34	0.000	4.832	204.037	0.000
November	59.08	0.000	5.126	36.12	0.000	5.126	196.197	0.000
December	66.29	0.000	6.107	37.53	0.002	6.107	168.848	0.000

**Table 3:** National scale metrics of CO_2_ emissions from buildings.

Month	Max CO_2_ (tons)	Ave CO_2_ (tons)	CO_2_/Vol (kg/m^3^)
January	3682	0.830	0.152
February	3348	0.770	0.141
March	2575	0.748	0.137
April	2300	0.587	0.108
May	2087	0.507	0.093
June	978	0.306	0.056
July	960	0.223	0.041
August	894	0.223	0.041
September	1215	0.350	0.064
October	1840	0.460	0.084
November	2389	0.545	0.100
December	2216	0.574	0.105

## Data Availability

The data used in this study are available through the links mentioned in the article.
